# Melatonin prevents senescence of canine adipose-derived mesenchymal stem cells through activating NRF2 and inhibiting ER stress

**DOI:** 10.18632/aging.101602

**Published:** 2018-10-25

**Authors:** Jia Fang, Yuan Yan, Xin Teng, Xinyu Wen, Na Li, Sha Peng, Wenshuai Liu, F. Xavier Donadeu, Shanting Zhao, Jinlian Hua

**Affiliations:** 1 College of Veterinary Medicine, Shaanxi Centre of Stem Cells Engineering & Technology, Northwest A&F University, Yangling, Shaanxi Province, China; 2 Department of Pathology, Yangling Demonstration Zone Hospital, Yangling, Shaanxi Province, China; 3 Division of Developmental Biology, The Roslin Institute Reader, Royal (Dick) School of Veterinary Studies University of Edinburgh, Easter Bush, Midlothian, EH25 9RG Scotland, UK

**Keywords:** canine adipose-derived mesenchymal stem cells, senescence, melatonin, endoplasmic reticulum stress, Nrf2, transplantation

## Abstract

Transplantation of adipose-derived mesenchymal stem cells (ADMSCs) can aid in the treatment of numerous diseases in animals. However, natural aging during *in vitro* expansion of ADMSCs prior to their use in transplantation restricts their beneficial effects. Melatonin is reported to exert biorhythm regulation, anti-oxidation, and anti-senescence effects in various animal and cell models. Herein, by using a senescent canine ADMSCs (cADMSCs) cell model subjected to multiple passages *in vitro*, we investigated the effects of melatonin on ADMSCs senescence. We found that melatonin alleviates endoplasmic reticulum stress (ERS) and cell senescence. MT1/MT2 melatonin receptor inhibitor, luzindole, diminished the mRNA expression levels and rhythm expression amplitude of Bmal1 and Nrf2 genes. Nrf2 knockdown blocked the stimulatory effects of melatonin on endoplasmic reticulum-associated degradation (ERAD)-related gene expression and its inhibitory effects on ERS-related gene expression. At the same time, the inhibitory effects of melatonin on the NF-κB signaling pathway and senescence-associated secretory phenotype (SASP) were blocked by Nrf2 knockdown in cADMSCs. Melatonin pretreatment improved the survival of cADMSCs and enhanced the beneficial effects of cADMSCs transplantation in canine acute liver injury. These results indicate that melatonin activates Nrf2 through the MT1/MT2 receptor pathway, stimulates ERAD, inhibits NF-κB and ERS, alleviates cADMSCs senescence, and improves the efficacy of transplanted cADMSCs.

## Introduction

Adipose-derived mesenchymal stem cells (ADMSCs) can self-renew and are multipotent being reportedly able to differentiate into multiple cell types, such as adipocytes, chondrocytes, osteoblasts, neuronal cells, and myocytes [1]. ADMSCs transplantation has already proven effective against severe traumatic, autoimmune, metabolic, and degenerative diseases. However, ADMSCs, similar to other types of mesenchymal stem cells (MSCs), cannot expand infinitely *in vitro* [2,3]. Senescence during *in vitro* culture reduces the quality and clinical efficacy of ADMSCs.

Melatonin is an endogenous indoleamine synthesized from tryptophan. Melatonin is produced by the pineal gland from where it is released into blood system circulation, and regulates numerous physiological and endocrine functions. An important function of melatonin is the regulation of biological rhythms [4]. The decline in melatonin secretion with age suggests it may have anti-aging functions. Melatonin regulates biological rhythms by controlling the expression of *Clock, Bmal1, Per 1-3,* and *Cry 1-2* [5].

In addition to regulating biorhythms, melatonin can also play an anti-aging role due to its antioxidant effects [6]. Melatonin directly removes reactive oxygen species (ROS), and its precursors and metabolites also have radical scavenging activity [7,8]. In addition, melatonin activates numerous antioxidant genes and promotes Nrf2 translocation [9]. NRF2 turns on the expression of several antioxidant and detoxification enzymes by binding to the antioxidant response element (ARE) in their promoter regions. Oxidative stress and other factors can activate NRF2 dissociation from KEAP1 and its nuclear translocation to function as a transcription factor. Numerous studies have shown that NRF2 is an essential regulator of longevity [10]. However, activation of NRF2 induces cellular senescence in fibroblasts [11]. This suggests that time-controlled activation of NRF2 may be critical for homeostasis in multicellular organism.

Melatonin has an anti- endoplasmic reticulum stress (ERS) effect in liver [12], nervous system [13], and lung diseases [14]. In Alzheimer’s disease melatonin improves cognitive function by inhibiting ERS. Chronic ERS is closely associated with tissue aging. The unfolding protein response (UPR), a cellular stress response related to ERS, also increases dramatically with aging [15–17].

The anti-senescence functions of melatonin on stem cells remain unclear. Several studies reported that melatonin reverses senescence via changes in SIRT1-dependent pathway, energy metabolism, epigenetic modifications, autophagy, circadian rhythm or other pathways [18,19]. However, whether replicative aging of canine ADMSCs (cADMSCs) is associated with ERS and whether melatonin has anti-ERS effects on cADMSCs remain unclear. In this study, we investigated the phenotype induced upon replicative aging of cADMSCs as well as the anti-senescent mechanism of melatonin in these cells.

## RESULTS

### Melatonin treatment relieves culture-induce senescence of cADMSCs

**Figure 1:**
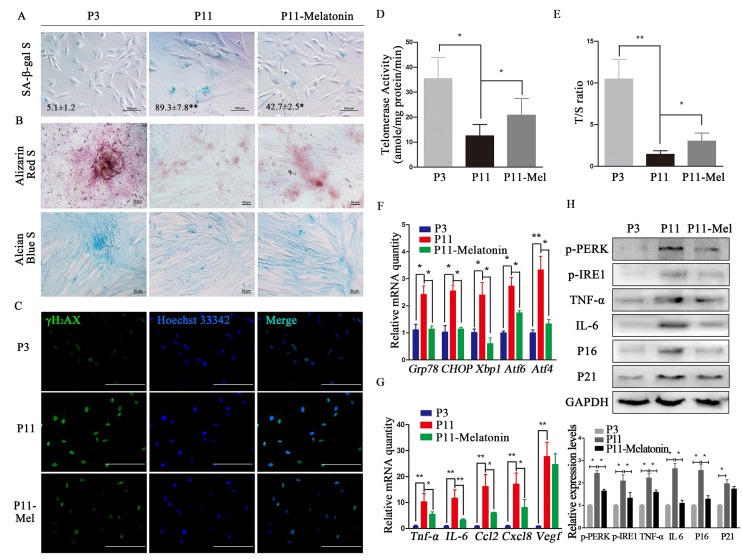
**Melatonin attenuates ERS and SASP in cADMSCs.** (**A**) SA-β-gal S of cADMSCs. (P3, 3rd passage, P11, 11th passage, P11–melatonin, melatonin-treated 11th passage) bar = 100 μm. (**B**) Alizarin Red and alcian blue staining of osteogenic and chondrogenic differentiation of cADMSCs. bar = 50 μm. (**C**) Immunocytochemistry of γH2AX in cADMSCs. bar = 200 μm. (**D**) Telomerase activity of cADMSCs. (**E**) Relative telomere length of cADMSCs. (**F**) Relative levels of SASP-related transcripts in cADMSCs. (**G**) Relative levels of ERS-related transcripts in cADMSCs. (H) Western blot quantification of ERS-related proteins (p-PERK and p-IRE1), SASP-related proteins (TNF-a and IL6), and senescent markers (P16 and P21).

Changes in cADMSCs morphology were apparent during prolonged *in vitro* culture. Staining for senescence-associated β-galactosidase (SA-β-gal S) increased between the 3^rd^ and 11^th^ passages. However, treatment with 1 μM melatonin for 7 d reduced the senescence phenotype of the three cADMSCs lines tested, as indicated by significantly reduced staining in cADMSCs at passage 11 treated with 1 μM compared to 0 μM melatonin (Fig. 1A). Therefore, 1 μM was chosen as the optimal concentration of melatonin to be used in subsequent experiments (Supplementary Figure 1).

The osteogenic and chondrogenic differentiation potential of cADMSCs decreased between the 3^rd^ and 11^th^ passages, but less so in melatonin-treated cADMSCs (Fig. 1B). Similarly, staining for γH2AX increased while telomerase activity and relative telomere length T/S ratio decreased between the 3^rd^ and 11^th^ passages, and these effects were attenuated by melatonin treatment (Fig. 1C-E). Moreover, transcript levels of SASP (*Ccl2, Tnf-a, Vegf, IL6* and *Cxcl8*) and ERS (*Grp78, Chop, Xbp1, Atf4* and *Atf6*) markers as well as protein levels of ERS (p-PERK and p-IRE1), SASP (IL6 and TNF-a), and senescent (P16 and P21) markers all increased in cADMSCs between the 3^rd^ and 11^th^ passages, and these effects were attenuated by melatonin treatment (Fig. 1F- H).

### ERS regulates senesce of cADMSCs

**Figure 2:**
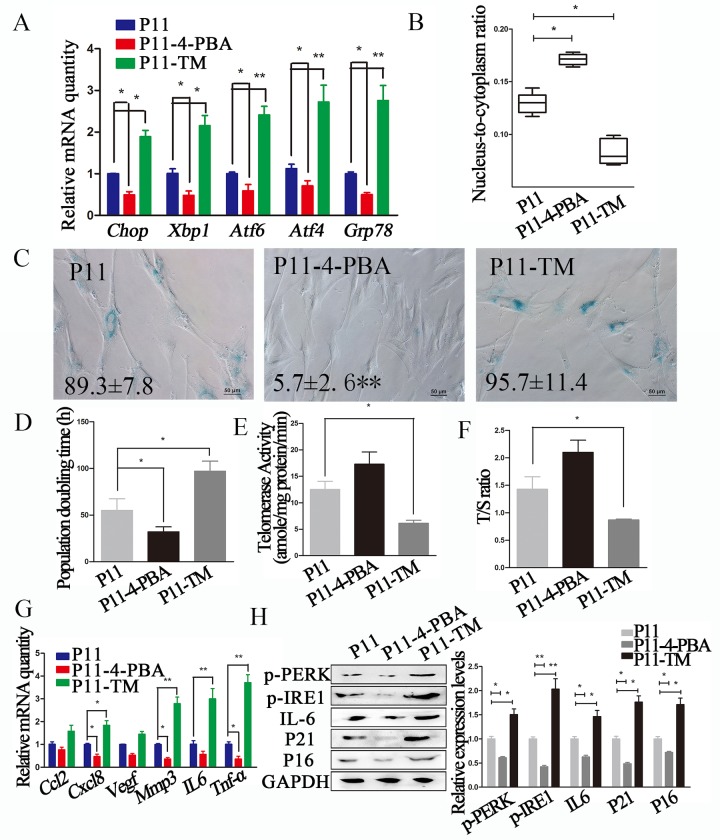
**ERS inhibitor 4-PBA attenuated the senescent phenotype in cADMSCs.** (**A**) Relative levels of of ERS-related transcripts in 4-PBA or TM-treated cADMSCs. (**B**) Nucleus-to-cytoplasm ratio of 4-PBA or TM-treated cADMSCs. (**C**) SA-β-gal S of control, 4-PBA-treated and TM-treated cADMSCs. bar = 50 μm. (**D**) Doubling time of 4-PBA or TM-treated cADMSCs. (**E**) Telomerase activity of cADMSCs. (**F**) Relative telomere length of cADMSCs. (**G**) Relative levels of of SASP-related transcripts in 4-PBA or TM-treated cADMSCs. (**H**) Western blot quantification of ERS-related proteins (p-PERK and p-IRE1), SASP-related protein (IL6), and senescent markers (P16 and P21).

To explore the relationship between the anti-senescent and ERS reducing effects of melatonin, cADMSCs at passage 11 were treated with either ERS inhibitor 4-PBA or ERS activator tunicamycin (TM). 4-PBA relieved the senescent phenotype of cADMSCs. Specifically, treatment with 0.25 mM 4-PBA for 12 h reduced the expression of ERS markers (*Grp78, Chop, Xbp1, Atf4* and *Atf6*) (Fig. 2A).

In addition, 4-PBA-treated cADMSCs showed a higher nucleus-to-cytoplasm ratio (Fig. 2B), lower SA-β-gal S staining (Fig. 2C), shorter population doubling time (Fig. 2D), and lower expression of SASP (*Cxcl8, Mmp3* and *Tnf-a*) markers (Fig. 2G) than non-treated cells. 4-PBA treatment also decreased the protein levels of ERS markers including p-PERK and p-IRE1, the SASP marker IL6, and the senescent markers P16 and P21 (Fig. 2H).

To confirm the association between reduced senescence and reduced levels of ERS markers, cADMSCs were treated with ERS activator TM (30 ng/mL for 12 h). TM increased the expression of ERS markers (*Grp78, Chop, Xbp1, Atf4* and *Atf6*) (Fig. 2A), reduced the nucleus-to-cytoplasm ratio (Fig. 2B), increased SA-β-gal S staining (Fig. 2C), increased the population doubling time (Fig. 2D), reduced telomerase activity (Fig. 2E) and the relative telomere length T/S ratio (Fig. 2F), and decreased the mRNA levels of SASP markers (*Il6, Cxcl8, Tnf-a* and *Mmp*3) (Fig. 2G) and protein levels of ERS (p-PERK and p-IRE1), SASP (IL6) and senescent (P16 and P21) markers (Fig. 2H).

### Melatonin activated circadian clock genes and NRF2, and decreased ERS through MT1/MT2

**Figure 3:**
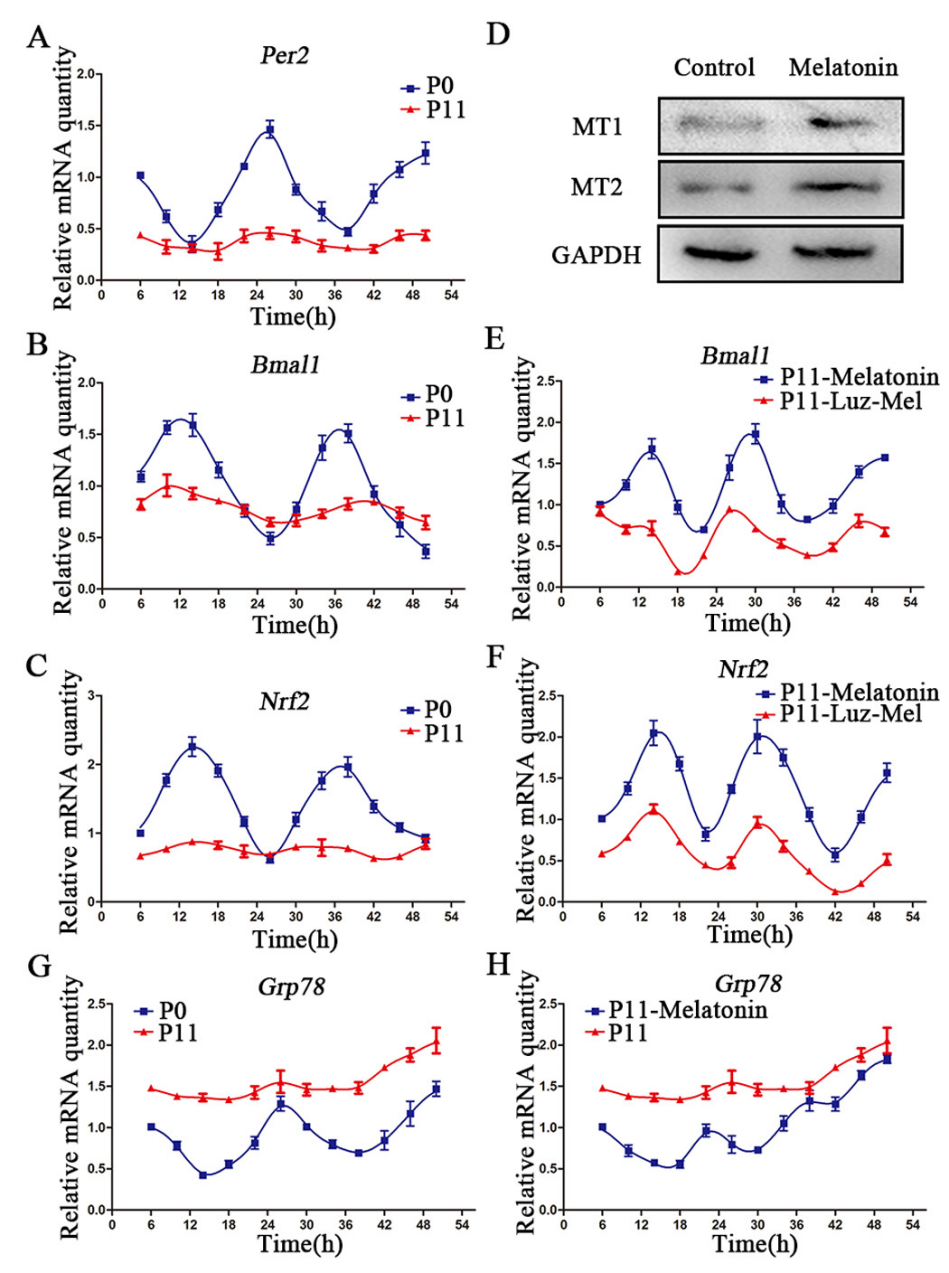
**Melatonin promotes rhythmic expression of Nrf2.** (**A**-**C**) Relative levels of Per2 (**A**) Bmal1 (**B**) and Nrf2 (**C**) in P0 and P11 cADMSCs. (**D**) Western blot quantification of MT1 and MT2 in control and melatonin-treated cADMSCs. (**E**-**F**) Relative levels of Bmal1 (**E**) and Nrf2 (**F**) in melatonin- and luzindole+melatonin-treated cADMSCs. (**G**-**H**) Relative levels of *Grp78* in P0 and P11 (**G**) and melatonin-treated P11 (**H**) cADMSCs.

Melatonin influences the body’s circadian clock as well as MSCs activity *in vitro* by regulating clock genes [20,21]. In addition, primary cell cultures can gradually lose their circadian rhythmicity. To further elucidate the anti-aging and circadian-regulatory effects of melatonin, we determined the expression of clock genes in primary cADMSCs. Cells at passage 0 exhibited higher amplitude circadian fluctuations of clock genes (Per2 and Bmal1) than cells at passage 11 (Fig. 3A-B).

NRF2 has been reported to be an important redox-sensitive and anti-aging transcription factor [22], and to be transcriptionally activated by clock genes via the E-box element [23]. Circadian fluctuations of Nrf2 in passage 0 cells were no longer detectable at passage11 (Fig. 3C). Similarly, circadian fluctuations of the ERS gene Grp78 in primary cADMSCs were lost during in vitro culture (Fig. 3G). Interestingly, melatonin treatment was able to restore fluctuations in the clock genes, *Bmal1* and *Nrf2,* in 11th passage cADMSCs (Fig. 3E-F), and decreased *Grp78* expression (Fig. 3H). In addition, melatonin treatment for 12 h increased protein levels of MT1/MT2 (Fig. 3D). Finally, the stimulatory effects of melatonin on gene expression fluctuations were inhibited by addition of the melatonin receptor inhibitor luzindole (1μM) (Fig. 3E-F). These results indicate that melatonin may activate rhythmic genes (e.g., NRF2) and inhibit ERS genes in cADMSCs through a receptor-mediated mechanism.

### Melatonin attenuated senescence of cADMSCs by activating NRF2

**Figure 4:**
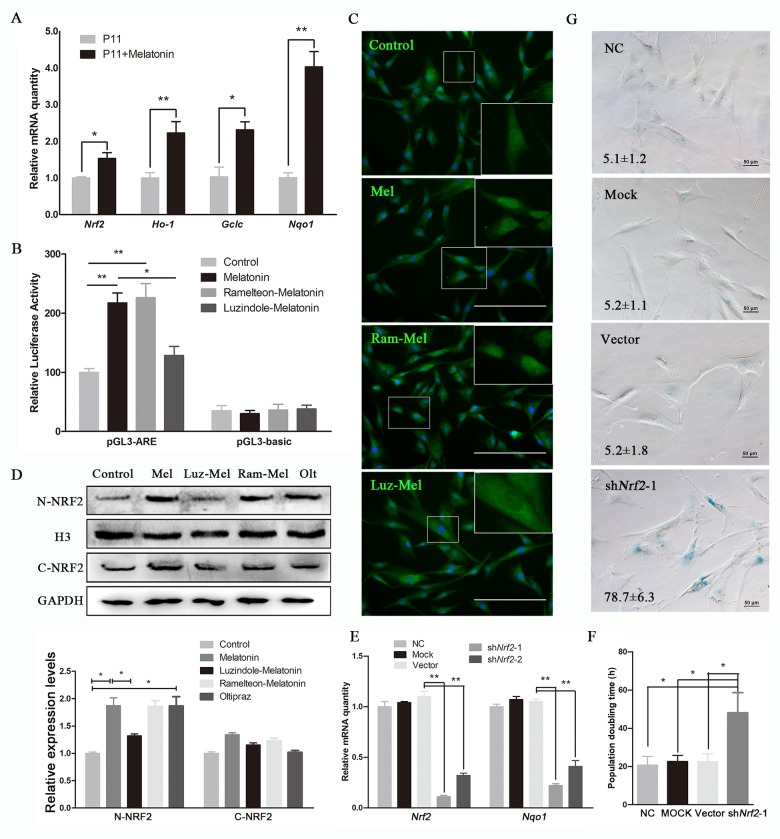
**Melatonin attenuates senescence of cADMSCs through activating NRF2.** (**A**) Relative levels of Nrf2, Ho-1, Gclc, and Nqo1 transcripts in melatonin-treated cADMSCs. (**B**) Dual-luciferase assay of melatonin-, ramelteon+melatonin-, and luzindole+melatonin-treated P11 cADMSCs. (**C**) Immunohistochemistry of NRF2 in control, melatonin-, ramelteon+melatonin- and luzindole+melatonin-treated P11 cADMSCs. bar = 200 μm (**D**) Western blot quantification of nucleoprotein and cytosolic proteins in melatonin-, ramelteon+melatonin- and luzindole+melatonin-treated cADMSCs, with oltipraz treatment as positive control. Oltipraz (15μM), an activator of NRF2, was used as the positive control. (**E**) Expression of Nrf2 and its target genes in P3 negative control, mock, vector, shNrf2-1, and shNrf2-2 cADMSCs. (**F**) Doubling time of P3 negative control, mock, vector and shNrf2-1 cADMSCs. (**G**) SA-β-gal S of P3 negative control, mock, vector and shNrf2-1 cADMSCs. bar = 50 μm.

Melatonin treatment for 12h increased the expression of *Nrf2* and its target genes, namely, *Nqo1*, *Ho-1*, and *Gclc* in 11^th^ passage cADMSCs (Fig. 4A). Dual-luciferase assay indicated that melatonin and MT1/MT2 activator ramelteon (10 nM) induced the transcriptional activity of NRF2, whereas luzindole (1μM) treatment inhibited this (Fig. 4B). Immunocytochemistry showed that NRF2 had a more intense nuclear staining in melatonin-treated and melatonin receptor agonist ramelteon-treated cADMSCs than in control and luzindole-treated cADMSCs. In contrast, cytoplasmic staining of NRF2 was more intense in control and luzindole-treated cADMSCs than in melatonin-treated and ramelteon-treated cADMSCs (Fig. 4C). Western blotting showed that NRF2 protein was increased in melatonin-treated cells or cells treated with a combination of melatonin and ramelteon (Fig. 4D). We then knocked down *Nrf2* in passage 3 cADMSCs via shRNA vector. This strategy resulted in 88% and 68% reduction in Nrf2 levels in two cell lines (shNrf2-1 and shNrf2-2), respectively (Fig. 4E), and led to an increase in the population doubling time (Fig. 4F) and SA-β-gal S staining (Fig. 4G) of melatonin treated cADMSCs. Thus, NRF2 mediated the anti-senescence effects of melatonin in cADMSCs

### Melatonin reduced ERS by activating NRF2-endoplasmic reticulum-associated degradation (ERAD)

**Figure 5:**
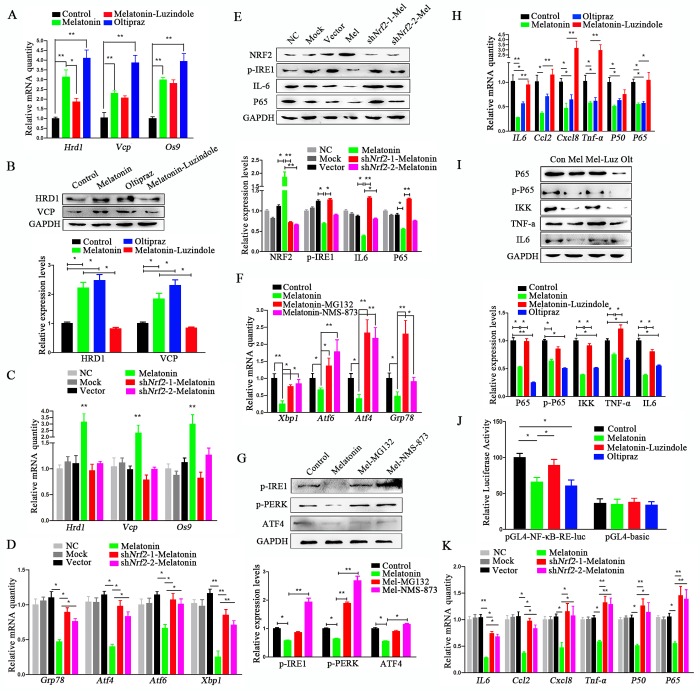
**Melatonin reduces ERS through activating NRF2 and ERAD and inhibiting NF-κB and SASP.** (**A**) Relative transcript levels of ERAD-related genes in P11 control, melatonin-, luzindole+melatonin- and oltipraz-treated cADMSCs. (**B**) Western blot quantification of ERAD-related protein in control, melatonin-, luzindole+melatonin- and oltipraz-treated P11 cADMSCs. (**C**) Relative transcript levels of ERAD-related genes in negative control, mock, shRNA vector, melatonin-treated, shNrf2-1, and shNrf2-2 P3 cADMSCs. (**D**) Relative transcript levels of ERS-related genes in negative control, mock, vector, melatonin-treated mock, shNrf2-1, and shNrf2-2 P3 cADMSCs. (**E**) Western blot and multiple quantifications of ERS-related protein (NRF2) in the negative control, mock, vector, melatonin-treated mock, shNrf2-1, and shNrf2-2 P3 cADMSCs. (**F**) Relative transcript levels of ERS-related genes in control, melatonin-, MG-132+melatonin- and NMS-873+melatonin-treated P11 cADMSCs. (**G**) Western blot quantification of ERS-related genes in control, melatonin-, MG-132+melatonin- and NMS-873+melatonin-treated P11 cADMSCs. (**H**) Relative transcript levels of SASP-related genes (P50 and P65) in control, melatonin-, luzindole+melatonin- and oltipraz-treated cADMSCs. (**I**) Western blot quantification of SASP-related proteins (P65, p-P65, and IKK) in control, melatonin-, luzindole+melatonin- and oltipraz-treated P11 cADMSCs. (**J**) NF-κB activity in control, melatonin-, luzindole+melatonin- and oltipraz-treated P11 cADMSCs was detected using dual-luciferase assay (**K**) Relative transcript levels of SASP-related genes (P50 and P65) in negative control, mock, vector, melatonin-treated mock, shNrf2-1, and shNrf2-2 P3 cADMSCs.

The reduction in ERS by melatonin through activation of ERAD has been reported in several studies [24–26]. Consistent with this, the ERAD markers *Hrd1, Vcp*, and *Os9* increased remarkably after treatment of cADMSCs with melatonin for 12h, while *Hrd1* was inhibited by luzindole (Fig. 5A). To test whether the ERAD-activating effect was related to NFR2 activity, we evaluated the expression of ERAD markers at 12h after treatment with the NRF2 activator oltipraz (15 μM). This resulted in the increased expression of ERAD marker to levels similar to those induced by melatonin treatment (Fig. 5A), a result that was confirmed by Western blot analyses (Fig. 5B). However, melatonin did not increase the expression of ERAD (*Hrd1, Vcp*, and *Os9*) (Fig. 5C) and ERS (*Xbp1, Atf4, Atf6*, and *Grp78*) markers (Fig. 5D) in shNRF2-cADMSCs. Levels of p-IRE1 and IL6 protein increased in shNRF2-cADMSCs compared with those in melatonin-treated cADMSCs (Fig. 5E). The ERS-reducing effect of melatonin treatment was blocked by ubiquitination and VCP specific inhibitors MG132 (20 μM) and NMS-873 (0.5 μM) (Fig. 5F-G). These results indicated that melatonin decreases ERS by activating ERAD.

### Melatonin reduced SASP by activating NRF2 and inhibiting NF-κB

SASP is deemed to be a trigger of ERS in aging cells [27]. We found that melatonin reduced the expression levels of SASP and ERS markers at 12h after treatment in 11^th^ passage cADMSCs (Fig. 1F-H). The NF-κB pathway is well-known to regulate SASP, so we tested the effect of melatonin treatment on NF-κB. Melatonin or oltipraz treatment for 12h decreased the transcript levels of P65 and P50 (Fig. 5H) and the protein levels of IKK, p-P65, and P65 (Fig. 5I). However, luzindole blocked the inhibitory effects of melatonin on NF-κB, and when compared to melatonin alone, luzindole in combination with melatonin treatment increased transcript levels of *P65, P50, IL6, Tnf-a, Ccl2*, and *Cxcl8* (Fig. 5H) and protein levels of IKK, p-P65, and P65 (Fig. 5I).

In addition, dual-luciferase assay showed that melatonin reduced the transcriptional activity of P65 (Fig. 5J). To confirm the NF-κB-reducing effect of NRF2, we determined the expression levels of *P65, P50, IL6*, and *Tnf-a* in shNRF2-cADMSCs, and showed that these increased significantly compared to 3^rd^ passage cADMSCs (Fig. 5K). The protein level of P65 and IL6 also increased in shNRF2-cADMSCs (Fig. 5E).

### Melatonin pretreatment increased the clinical efficacy of cADMSCs

**Figure 6:**
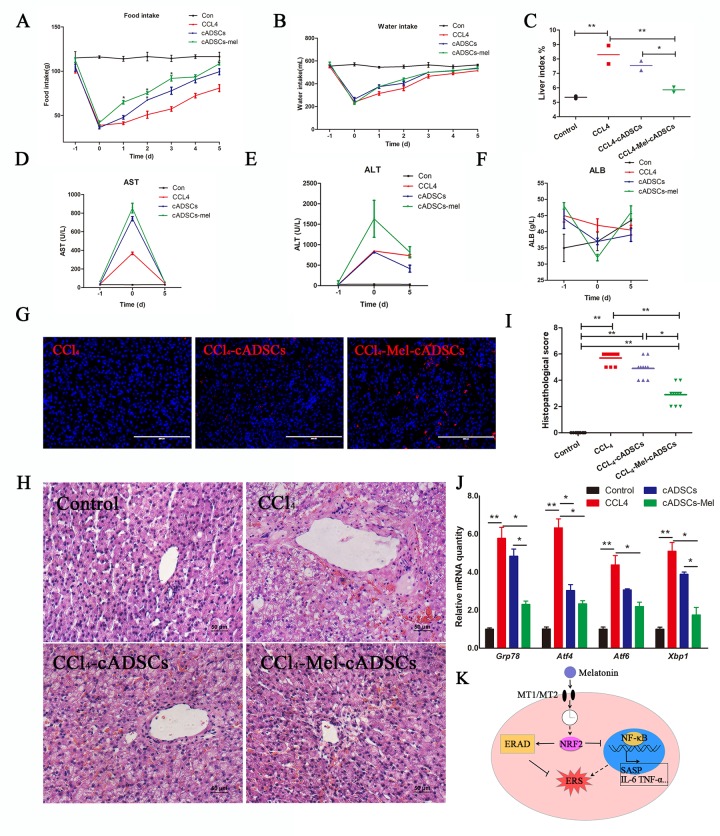
**Melatonin pretreatment increases the survival rate and curative effect of cADMSCs transplantation.** (**A**) Food intake, (**B**) water intake, and (**C**) liver index of experimental dogs. (**D**-**F**) Effect of CCl_4_ injection and cADMSCs transplantation on blood AST (**D**), ALT (**E**) and ALB (**F**). (**G**) Frozen liver sections obtained 5 days after cADMSCs transplantation. Bar = 200 μm (**H**) HE staining of liver sections from CCl_4_-injected dogs 5 days after cADMSCs transplantation. Bar = 50 μm. (**I**) Histopathological scores of HE-stained canine liver sections. (**J**) Effects of CCl_4_ injection and cADMSCs transplantation on the levels of ERS-related transcripts in canine liver. (**K**) Proposed model of melatonin inhibition of ERS. According to this model, melatonin binding to MT1/MT2 receptor results in activation of NRF2 which then activates ERAD and inhibits NF-κB signaling, overall resulting in inhibition of ERS. A dotted line indicates information obtained from other studies.

cADMSCs have been reported to aid regeneration of injured liver [28], however, the therapeutic properties of cADMSCs may be reduced by long-term culture in vitro [29]. To explore the effects of melatonin on the therapeutic potential of cADMSCs, we transplanted cADMSCs, previously treated or not with melatonin, into CCl4-treated dogs, a common model of induced acute liver injury. cADMSCs (1× 10^7^ per 10 mL) were administered by intravenous injection to dogs 10 h after administration of CCl4. cADMSCs at passage 9 rather than passage 11 were used as we reasoned that higher cell viability would resulted in higher therapeutic effects. Food and water intake analyses showed that cADMSC injection accelerated recovery relative to CCL4-induced acute liver injury dogs. Average food intake of melatonin-pretreated cADMSCs injection group was significantly higher than CCL4 injury group from the 1st day after cADMSCs transplantation (Fig. 6A-B). Hepatic tissue was collected on the 5^th^ day after cell transplantation. The liver index (liver/body weight) of the CCl_4_ group was significantly higher than that of the control group, and this increase was prevented by injection with melatonin-pretreated cADMSCs (Fig. 6C). Blood serum was collected 1 day before CCL_4_ injury, 10 h after CCl_4_ injection and on the 5^th^ day after cell transplantation. Aspartate aminotransferase (AST) and alanine aminotransferase (ALT) significantly increased, whereas Albumin (ALB) decreased after CCl_4_ injection. Higher recovery rates were observed in dogs injected with melatonin-pretreated cADMSCs than with untreated cADMSCs. (Fig. 6D-F). PKH26-positive cells were found in frozen liver sections of cADMSCs-injected dogs. Red fluorescence intensity was higher in tissues from dogs injected with melatonin-pretreated cADMSCs than with untreated cADMSCs (Fig. 6G). HE staining of liver sections showed extensive histopathological changes induced by CCl_4_, characterized by hepatic lobule impairment, severe hepatocyte degeneration, necrosis, fatty changes, inflammatory cell infiltration, and congestion (Fig. 6H). Histopathological scores for acute liver injury are shown in Supplementary Table 1. Five different visual fields from 2 donors’ liver sections were analyzed in each group. Tissues from dogs transplanted with melatonin-pretreated cADMSCs had a significantly smaller score than those from dogs transplanted with untreated cADMSCs (Fig. 6I). Expression of ERS-related genes *Grp78,* *Atf4, Atf6*, and *Xbp1* was increased by CCl_4_ treatment, and this effect was attenuated in animals transplanted with melatonin-pretreated cADMSCs relative to untreated cADMSCs (Fig. 6J).

## DISCUSSION

The therapeutic value of ADMSCs has been shown in numerous studies [30]. Properties such as easy sourcing free of ethical concerns and lack of tumorigenicity render ADMSCs an ideal cell source for regenerative therapies. However, ADMSC senescence during in vitro expansion prior to transplantation reduces the survival rate and therapeutic efficacy of these cells. Cell morphology, proliferation rate, differentiation potential, and gene expression change during repeated passages of ADMSCs [2]. The SASP of senescence cells even contributes to systemic dysfunction in age-related diseases [31]. In this study, the expression levels of SASP- and ERS-related transcripts and proteins increased in senescent cADMSCs. Reports on the relationship between cell senescence and ERS are scarce and often disagree [15–17,32]. Also, the ERS inhibitor 4-PBA attenuates aging of bone marrow-derived MSCs in patients with systemic lupus erythematosus [33].

Melatonin has anti-aging actions in a number of animal and cell models [34]. For example, melatonin attenuated a reduction in telomerase activity in the retina of patients with age-related macular degeneration [35], as well as senescence of bone marrow MSCs [36]. We showed that the inhibitory effects of melatonin on cell senescence involved effects on ERS. Melatonin mitigates memory deficits [37] and Alzheimer-like damage [38] through alleviating ERS. Melatonin also shows ERS suppressive effects in numerous other diseases [12–14].

The mechanisms involved in these effects are unclear. NRF2 is a transcription factor that regulates various anti-oxidation and detoxification enzymes. NRF2 has been reported to prolong the life span of mice or *Caenorhabditis elegans* [22,39]. Melatonin can activate NRF2 by inhibiting its ubiquitination [40] and promoting its transportation to the nucleus [41]. Herein, we showed a link between the activation of NRF2 by melatonin and circadian clock gene activity. Melatonin not only alters the transmission effects of biological clock, but also acts as a *zeitgeber* which stabilizes, strengthens, and coordinates biological rhythmicity. Melatonin acts on the MT1/MT2 receptor of the suprachiasmatic nucleus, pituitary, brown fat, pineal gland, and other tissue cells and regulates the rhythmic expression of *Clock, Bmal, Per, and Cry* [20]. A previous study showed that Bmal1 and Clock in broncho alveolar epithelial cells can bind to the E-box (5'-CACGTG-3') of target genes including Nrf2 and regulate the transcription of numerous rhythmic genes [23]. A similar E-box sequence (5’-GACGTG-3’) exists in the promoter region of the *Nrf2* gene in canines. We found that, despite melatonin increasing Bmal1 and Nrf2 fluctuations in cADMSCs at passage 11, expression of these genes was not restored to the levels observed in primary passage of cADMSCs. This may be due to melatonin treatments were transient rather than involving a slow and prolonged increase as occurs *in vivo.* We found that melatonin also regulates the rhythmic expression of the ERS-related gene *Grp78*. However, further studies on the melatonin-induced rhythm expression of ERS-related genes are necessary.

ERAD promotes the degradation of misfolded proteins, prevents protein aggregation in the endoplasmic reticulum, and protects cells against chronic ERS. In the current study, melatonin had an anti-ERS effect upon activation of the ERAD pathway in cADMSCs. Recent reports have shown that melatonin reduces ERS in corneal fibroblasts by activating ERAD [42]. Several studies have shown that NRF2 is a master regulator of ERAD-related genes [43]. The activity of protease increased and aging was effectively alleviated in NRF2 activator-treated human skin fibroblasts [44]. Herein, NRF2 and ERAD activation were found to be indispensable for the anti-ERS action of melatonin-treated cADMSCs.

NF-κB is an important regulation of the expression of SASP-related genes in senescent cells [45]. SASP is a major cause of cell UPR in senescent cells, because increased secretion of SASP-related factors leads to the accumulation of excessive unfolded proteins in the endoplasmic reticulum [27]. This phenomenon has been confirmed in chemotherapeutic drug-induced senescence. Studies have shown that UPR reactions occurs only in senescent cells with SASP [46]. Oxidative stress activates NF-κB [47]. As a major regulator of antioxidant genes, Nrf2 and its activator can also inhibit the NF-κB pathway [48]. However, the mechanisms by which Nrf2 and NF-κB interact require further study.

The mechanisms by which cADMSC injection is beneficial to animals with liver injury is unclear. Some studies suggested that MSCs can differentiate into hepatocytes thereby reconstructing damaged liver tissue [49]. Other studies demonstrated that MSCs secrete growth factors and other substances that promote the self-reconstruction of liver tissues [50]. Yet others showed that MSCs act by reducing inflammatory responses [51]. In our study, melatonin pretreatment improved the survival of cADMSCs and decreased ERS in the acute liver injury model. These results suggest that melatonin treatment inhibited senesce of cADMSCs and improved their beneficial actions after transplantation.

Overall, our results show that melatonin had an anti-senescent effect in cADMSCs by inhibiting ERS through activation of rhythmic expression of NRF2, activating the ERAD pathway, and inhibiting the NF-κB pathway (Fig. 6K). Melatonin treatment improved the survival rate of cADMSCs in the acute liver injury model.

## MATERIALS AND METHODS

### Cell isolation, identification, and culture

cADMSCs [52] were isolated by collagenase type I (Roche Diagnostics, Switzerland) digestion of abdominal subcutaneous adipose tissue collected from three 1 year old female cross-bred dogs. cADMSCs were cultured in α-MEM (Invitrogen, Carlsbad, CA) supplemented with 10% FBS (HyClone, UT, USA), 2 mM L-glutamine, and 1% non-essential amino acids (Invitrogen) in a humid atmosphere of 5% CO_2_ at 37 °C. Cells were passaged every 2 days with trypsin–EDTA (Invitrogen). The identity of ADMSCs was confirmed by flow cytometry and differentiation into adipogenic lineage and osteogenic lineage, as in previous studies [52]. Melatonin (M5250; Sigma, Milan, Italy) was dissolved in dimethyl sulfoxide (DMSO; D5879; Sigma) at a concentration of 10 mM. DMSO only was used as control cells.

### Osteogenic and chondrogenic differentiation of cAMSCs in vitro

About 2×10^4^ cells were seeded into 12-well plates in αMEM with 10% FBS, 100 nmol/L dexamethasone, 30 μg/mL ascorbic acid, and 10 mmol/L β-glycerophosphate (Sigma-Aldrich, St. Louis, MO, USA) for 14 days. Osteogenic differentiation was assessed by alizarin red staining. To induce chondrogenesis, cADMSCs were grown in αMEM with 10% FBS, 40 ng/mL dexamethasone, 50 μg/mL ascorbic acid, 50 μg/mL L-proline, 1 mM sodium pyruvate (all Sigma-Aldrich), insulin–transferrin–selenium X (Gibco, Carlsbad, California, USA), and 10 ng/mL transforming growth factor-β3 (PeproTech, Rocky Hill, NJ, USA) for 14 days. Chondrogenesis was assessed with alcian blue staining.

### Senescence associated β-galactosidase staining

Cells were stained with β-galactosidase staining kit (Beyotime, Shanghai, China). cADMSCs were fixed for 15 min. The cells were washed 3 times with PBS followed by staining with the solution A, B, C and X-gel mixed liquor for 10 h at 37 °C.

### Population doubling time (PDT)

The population doubling time (PDT) of cADMSCs was estimated according to the formula *PDT = [log 2/(log Nt−logN0)]× t*. *N0* indicates the number of seeded cells, *Nt* indicates the number of cells after t h of culturing, and *t* refers to the duration of cell culture in hours.

### Nucleocytoplasmic ratio

Cells were fixed in 4% paraformaldehyde in phosphate-buffered saline (PBS) at room temperature (RT) for 10 min. Nuclear staining was performed with 1 μg/mL Hoechst 33342 (Sigma Aldrich). Fluorescence images was obtained with Evos f1 fluorescence microscope (AMG, USA) and analyzed using Image J software (National Institutes of Health, USA).

### Telomerase activity assay

Telomerase activity in cell extracts was measured using the TRAPeze RT Telomerase Detection Kit (S7710, Millipore, USA). The amount of extended telomerase substrate (amoles) produced per mg of protein per minute for each sample cell extract was obtained as per manufactuer’s instructions and used as Y-axis when drawing bar charts.

### Telomere length assays

cADMSCs were extracted using a DNA Isolation Kit (Tiangen, China) according to the manufacturer’s instructions. The ratio of telomere repeat copy number to single gene copy number (T/S) was determined using QRT-PCR in the CFX96 Real-Time PCR system. QRT-PCR procedures were described as follows: pre-denaturation at 94 °C for 10 min, followed by 39 cycles for 15 s at 94 °C, and annealing for 1 min at 56 °C. The telomere reaction mixture consisted of 1× Quantitect Sybr Green Master Mix, 2.5 mM of DTT, 100 nM of Tel-F primer (CGGTTTGTTTGGGTTTGGGTTTGGGTTTGGGTTTGGGTT), and 900 nM of Tel-R primer (GGCTTGCCTTACCCTTACCCTTACCCTTACCCTTACCCT). 36B4 was used as the loading control, with 36B4-F primer (ACTGGTCTAGGACCCGAGAAG) and 36B4-R primer (TCAATGGTGCCTCTGGAGATT). DNA quantitation was performed using Thermo NanoDrop 2000 (Thermo Scientific) and double dilution of DNA in the control sample. Comparative CT values from QRT-PCR were used to draw the standard curve. The T/S ratio for each sample was calculated by dividing of the average 36B4 ngDNA value by the average telomere ngDNA.

### Synchronization of cADMSCs

When cADMSCs reached 50% confluence they were treated with α-MEM medium containing 0.5% FBS for 24 h. The medium was then changed to α-MEM medium containing 50% FBS for 1h. cADMSCs were then cultured with α-MEM medium containing 0.5% FBS with or without melatonin during which samples were collected every 4 hours and the relative expression of genes was determined.

### Quantitative real-time PCR analysis

The total RNA of cADMSCs was extracted using Trizol reagent (Takara, Japan) according to the manufacturer’s instructions. Reverse Transcriptase Reagent kit (Thermo Scientific) was used according to the manufacturer’s instructions. Quantitative real-time PCR (QPCR) was carried out with the CFX96 Real-Time PCR system as follows: pre-denaturation at 94 °C for 5 min, following 39 cycles for 30 s at 94 °C, annealing for 30 s at 58 °C and 30 s at 70 °C for extending. Glyceraldehyde 3-phosphate dehydrogenase (GAPDH) was used as the loading control. Comparative CT-values from QPCR were used to measure relative gene expression. Primers are listed in Supplementary Table 2.

### Construction of the ShNrf2 interference vector

The plasmid pSIH-H1-CopGFP-shRNA was used. Target sequences were designed by BLOCK-iT™ RNAi Designer(Thermo Fisher) and are listed in Supplementary Table 3.

### Dual-luciferase assay

Plasmids pGL3-ARE-luc were used to analyze the NRF2 activity. pGL4-NF-κB-RE-luc (Promega, USA) was used to analyze the NF-κB activity. The pRL-TK Renilla luciferase (Promega, USA) plasmid was used to control for transfection efficiency. The activities of Firefy and Renilla luciferase were determined using the dual-luciferase reporter assay system (Promega, USA) according to manufacturer instructions. Assays were independently conducted at least in triplicate. The data presented show relative Firefy luciferase activity normalized to Renilla luciferase activity.

### Immunocytochemistry

Cells were fixed in 4% paraformaldehyde in phosphate-buffered saline (PBS) at room temperature (RT) for 10 min, washed three times with PBS, and then permeabilized for 15 min with 0.1% Triton-X 100 (Sigma-Aldrich, St. Louis, MO) in PBS at RT. Cells were blocked with PBS supplemented with 4% bovine serum albumin for 30 min and incubated with primary antibodies against NRF2 (1:200, D121053, Sangon Biotech, China), and γH2AX (1:200, sc-517348, Santa Cruz, USA) at 4 °C for 16 h. After washing with PBS three times, cells were incubated with secondary antibodies for 1 h at 37 °C in the dark. Following another three washing steps in PBS, nuclear counterstaining was performed with 1 μg/mL Hoechst 33342 (Sigma Aldrich). Fluorescence images were obtained by Evos f1 fluorescence microscope (AMG, USA).

### Western blot

Total cell extracts were prepared in 1× sodium dodecyl sulfate- polyacrylamide gel electrophoresis (SDS-PAGE) sample loading buffer. Cell fractions were extracted with nuclear and cytoplasm protein extraction kit (Wanleibio, China). Cell proteins were resolved by SDS-PAGE, transferred to a polyvinylidene difluoride membrane, and probed with GAPDH (1:1000, BM3876, Bosterbio, USA), p-PERK, p-IRE1, ATF4, P16 (1:500, bs-3330R, bs-16698R, bs-1531R, bs-20656R, Bioss, China), H3, NRF2 (1:500, D153567, D121053, Sangon Biotech, China), and TNF-α, IL-6, P21, MT1, MT2, HRD1, VCP, P65, p-P65, IKK (1:200, sc-52746, sc-32296, sc-136020, sc-13180, sc-13177, sc-293484, sc-136273, sc-514451, sc-166748, sc-7606, Santa Cruz, USA). Secondary anti-rabbit, anti-mouse antibodies (1:1000, BM2004, BA1001, Bosterbio, USA) conjugated with horseradish peroxidase were used. Detection was performed using a Thermo Scientific Pierce enhanced chemiluminescence western blotting substrate (Thermo Scientific). Results were analyzed by Tanon-410 automatic gel imaging system (Shanghai Tianneng Corporation, China).

### Acute hepatic injury model

All the animals were used according to Chinese Laboratory Animal Guidelines and after approval by the committee of Shaanxi Centre of Stem Cells Engineering & Technology, Northwest A&F University. Eight 1 year old female small cross-bred dogs with body weight 5 ± 0.1 kg were used. All animals were kept under constant temperature (25 ± 2°C) and light (12:12 h light:dark cycle) and granted free access to standard dry chow and water. The dogs were randomly assigned to four experimental groups (n = 2): control (intraperitoneal injection of 0.54 mL/kg olive oil,); CCl4 (intraperitoneal injection of 40% CCl4 dissolved in 0.9 mL/kg olive oil,); cADMSCs (intravenous injection of 100 million PKH26 (Sigma, USA)-labeled cADMSCs in 10 mL phosphate buffer saline (PBS) at 10 h after CCl4); and cADMSCs–melatonin (cADMSCs were pretreated with 1μΜ melatonin for 7d before injection to dogs). The liver index (liver weight (g)/body weight (g) × 100) was calculated 5 days after cADMSCs transplantation. Blood biochemistry was performed before and 10 h after CCl_4_ injection, and 5 days after cADMSCs transplantation. Aspartate aminotransferase (AST), alanine aminotransferase (ALT) and Albumin (ALB) activities in serum were analyzed by FUJI DRI-CHEM NX500iVC biochemical analyzer (FUJI Film, Japan). HE staining of frozen and paraffin sections was conducted at 5 days after cADMSCs transplantation. Supplementary Table 1 shows the histopathological score for acute liver injury. The histopathological score of 5 different visual fields from 2 donor liver was analyzed in each group.

### Statistical analysis

One-way ANOVA was used followed by Newman–Keuls multiple range tests whenever main effects were significant. Student's t-test was used when comparing two means. All data are presented as mean ±SD, and statistical significance is shown as follows: *p < 0.05; **p < 0.01; ***p < 0.001. All data were analyzed using GraphPad Prism software (La Jolla, CA, USA) and represent at a minimum of three different experiments.

## Supplementary Material

Supplementary Figure

Supplementary Tables
