# Supplementary material for: Melatonin prevents senescence of canine adipose-derived mesenchymal stem cells through activating NRF2 and inhibiting ER stress
Source: Aging (Albany NY). 2018 Oct 25;10(10):2954–72. doi: 10.18632/aging.101602 (PMC6224246; doi:10.18632/aging.101602)
Supplement: Supplementary Figure [file aging-10-101602-s001.pdf]

SUPPLEMENTARY FIGURE

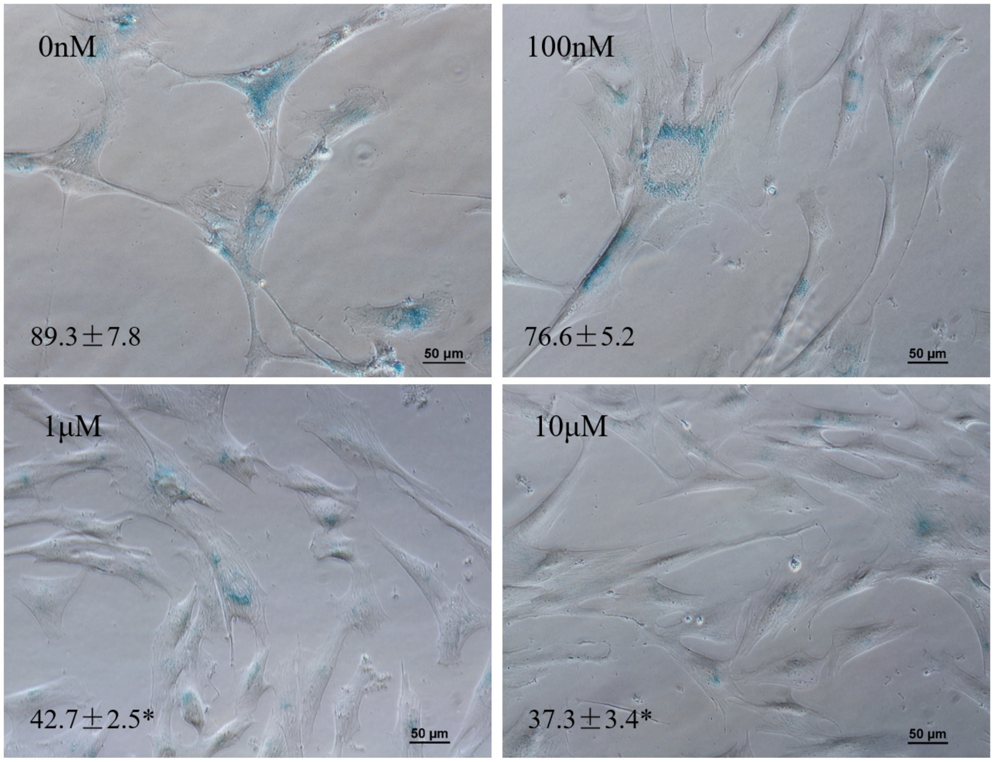

Supplementary Figure 1. The positive rate of SA-b-gal staining on P11 cADMSCs treated with different concentrations of melatonin. Bar = 50 μm.
