# Supplementary material for: Melatonin prevents senescence of canine adipose-derived mesenchymal stem cells through activating NRF2 and inhibiting ER stress
Source: Aging (Albany NY). 2018 Oct 25;10(10):2954–72. doi: 10.18632/aging.101602 (PMC6224246; doi:10.18632/aging.101602)
Supplement: Supplementary Tables [file aging-10-101602-s002.pdf]

SUPPLEMENTARY TABLES

Supplementary Table 1. Acute hepatic injury pathology score.

Score	Pathological manifestations
0	No abnormalities
1-2	Liver structure remains intact，a few scattered hepatocyte necrosis，regional mild hepatocyte degeneration.
3-4	Lobules of liver were disorder. Less than a third of the hepatic lobule was necrotic. The necrosis in the central area of the lobule is rare, and diffuse moderate hepatocyte degeneration.
5-6	Most of the liver structures were destroyed. Moderate and severe necrotic. Lobular center and bridge necrosis are widespread.

Supplementary Table 2. Primers used in this study.

Primers	Sequence (5'-3')
Xbp1-F	GCAGCACTCAGACTACGTGCATC
Xbp1-R	GATCCAAGTTGAACAGAATGCCC
Grp78-F	AGAAACTCCGGCGTGAGGTAGA
Grp78-R	TTCCTGGACAGGCTTCATGGTAG
Chop-F	GCCCTCACTCTCCAGATTCCA
Chop-R	CTGTGCCACTTTCCTCTCGTTC
Atf4-F	CCTGAGCAGCGAGGTGTTGG
Atf4-R	AGCCCGCCTTAGCCTTGTC
Atf6-F	ATCAGCGGGAACTCAGGGAGT
Atf6-R	TTGTGGTCTTGTTGTGGGTGGT
IL-6-F	TGATGGCTACTGCTTTCCCTACC
IL-6-R	CCAGTGCCTCTTTGCTGTCTTC
Cxcl8-F	ATGACTTCCAAGCTGGCTGTTG
Cxcl8-R	ATGTGGGCCACTGTCAATCACT
Mmp3-F	TGATGAACAATGGACAAGCGATAC
Mmp3-R	CTAGGGTCAGCCGAGTGAAAGAG
Ccl2-F	AGGTGCTCACCCAGCCAGAT
Ccl2-R	AGCTTCTTTGGGACACTTGCTG

Vegf-F	TGCCCAAGAAATCAACCCCTA
Vegf-R	TTGCTCACAATGCCTCAGTCG
Tnf-α-F	AAGGTCAACCTACTCTCTGCCATC
Tnf-α-R	CTGAGTCGATCACCCTTCTCCA
Per2-F	CCAGGTCACCAGATAAACACGC
Per2-R	TTGCTGATAGGATGGGCTATGC
Bmal1-F	GACAGCGAACCAGACAACGAG
Bmal1-R	CTTCCCATCTATTGCGTGCC
Nrf2-F	TGAGGATTCTTTCAGCAGCATC
Nrf2-R	GCCTTCAATAGTCCCGTCCA
Gclc-F	TGGTGTTTGTGGTGCTGCTTAC
Gclc -R	GTAGCCATCTACTACAGTGTTGCCAC
Ho-1-F	CTTCTTCACCTTCCCCAACATC
Ho-1-R	CTCCTCAAACAGCTGAATGTTCAG
Nqo1-F	CACACTCCTATGGATGTCCGAAT
Nqo1-R	AGTCAAAGAGGCTACTTGGAGCA
Hrd1-F	TGCCGCATTGTCTCCCTCAT
Hrd1-R	TCGCATACTCAAAGCCAAACACC
Vcp-F	GGAGGTGGAAAGGCGTATTGTAT
Vcp-R	GTCAATGCTATTGGGTCTGTTGG
Os9-F	AAGCAACATCGCCTGAAACG
Os9-R	GAAATACCAGCACCCTCGTCAC
P50-F	CAGATAGTTTCGGTGGCGGTA
P50-R	CTTCAGAATCATTTTTAGATGGGGT
P65-F	GAAGAACAGCGTGGGGACTATG
P65-R	TGTCAAAGATGGGATGGGAGAG
Gapdh-F	GCTGCCAAATATGACGACATCA
Gapdh -R	GTAGCCCAGGATGCCTTTGAG

Supplementary Table 3. Parameters of the short hairpin RNA (shRNA).

shRNA	Sequence (5'-3')
Sh*Nrf2*-1-S	GATCCGCAGCAGGACATTGAGCAATCAAGAGTTGCTCAATGTCCTGCTGCTTTTTTG
Sh*Nrf2*-1-A	AATTCAAAAAAGCAGCAGGACATTGAGCAACTCTTGATTGCTCAATGTCCTGCTGCG
Sh*Nrf2*-2-S	GATCCGTAAGAAGCCAGATATTAATCAAGAGTTAATATCTGGCTTCTTACTTTTTTG
Sh*Nrf2*-2-A	AATTCAAAAAAGTAAGAAGCCAGATATTAACTCTTGATTAATATCTGGCTTCTTACG
Scrambled shRNA-S	GATCCGATGAAATGGGTAAGTACATTCAAGAGATGTACTTACCCATTTCATCTTTTTTG
Scrambled shRNA-A	AATTCAAAAAAGATGAAATGGGTAAGTACATCTCTTGAATGTACTTACCCATTTCATCG
